# Estimating the population eligible for Medicare's obesity-coverage demonstration

**DOI:** 10.1093/haschl/qxag283

**Published:** 2026-10-19

**Authors:** Brian B Reid, Aidan P Sullivan, Sean D Sullivan

**Affiliations:** Reid Strategic, Scarborough, ME 04074, United States; Center for the Evaluation of Value and Risk in Health, Tufts University School of Medicine, Boston, MA 02111, United States; Englewood CO 80113, United States; The Comparative Health Outcomes, Policy, and Economics (CHOICE) Institute, University of Washington School of Pharmacy, Seattle, WA 98195, United States

**Keywords:** medicare, obesity, semaglutide, tirzepatide, CMS, diabetes, bridge, NHANES

## Introduction

On July 1, 2026, the US Centers for Medicare and Medicaid Services launched the Bridge demonstration program, which will make glucagon-like peptide-1 (GLP-1) receptor agonists for obesity available to certain eligible Medicare beneficiaries.

This represents a marked change in access to obesity medicines; under the federal law that established the Medicare Part D program, treatments for weight loss are not eligible for reimbursement. Key features of the Bridge program, including clinical eligibility, prior authorization criteria, and covered treatment options, are detailed in a prior publication.^1^ The program runs outside standard Part D plan rules.

Beneficiaries eligible for Bridge will pay $50 a month for medicines dispensed under the program, and the federal government will pay $195. CMS has not estimated the number of eligible patients. We fill that gap by applying the published Bridge criteria to the CDC's National Health and Nutrition Examination Survey (NHANES).

## Methods

### Data

We estimated the number of adults aged 65 years or older who would meet Bridge eligibility criteria using the 2017-2020 NHANES,^2^ a nationally representative survey of the civilian, noninstitutionalized US population. NHANES uses a complex, multistage, stratified probability sampling design. Estimates were weighted to the civilian, noninstitutionalized US population and accounted for NHANES's survey design using strata, primary sampling units, and phlebotomy weights.

### Bridge eligibility criteria

Bridge eligibility was operationalized as age ≥65 years and 1 of 3 BMI-based pathways: BMI ≥35 kg/m^2^; BMI ≥30 kg/m^2^ with heart failure, uncontrolled hypertension, or chronic kidney disease stage ≥3a; or BMI ≥27 kg/m^2^ with prediabetes, prior myocardial infarction, or prior stroke.

Prediabetes was defined as hemoglobin A1c 5.7% to <6.5%, and chronic kidney disease stage ≥3a was defined as estimated glomerular filtration rate <60 mL/min/1.73 m^2^ using the 2021 CKD-EPI creatinine equation.^3^ Symptomatic peripheral artery disease could not be directly implemented because the 2017-2020 NHANES dataset does not capture ankle-brachial index and includes no self-report question.

Heart failure was assessed using self-reported congestive heart failure, and uncontrolled hypertension was defined as measured systolic blood pressure ≥140 mmHg or diastolic blood pressure ≥90 mmHg among respondents reporting 2 or more current blood pressure medication use.

The Bridge eligibility criteria exclude patients with diabetes, obstructive sleep apnea (OSA), and metabolic dysfunction-associated steatohepatitis (MASH). Diabetes was applied as an exclusion using self-reported physician diagnosis or hemoglobin A1c ≥ 6.5%. OSA was incorporated into the base case through a conservative modeled adjustment excluding 20% of otherwise eligible adults with BMI ≥30 because OSA data are not included in NHANES 2017-2020; this assumption was based on a review^4^ reporting mean OSA prevalence of 22% in men and 17% in women across population-based studies. A 15% OSA exclusion and no OSA adjustment were evaluated as sensitivity analyses. MASH was assessed by excluding patients with a positive liver elastography finding.

Confidence intervals for OSA-adjusted estimates treated the OSA rate as fixed and reflect NHANES sampling uncertainty only.

The supplement contains information (Tables S1-S6) on construction and characteristics of the sample.

## Results

Based on a representative sample of the 2017-2020 65-and-older population who indicated they had Medicare coverage, which broadly reflects Medicare beneficiaries, the Bridge-eligible population is 10.93 million (95% CI 9.40 million to 12.47 million) when an OSA rate of 20% is assumed (Table 1). A lower OSA sensitivity assumption of 15% yields an estimate of 11.39 million, while no OSA adjustment yields an upper-bound estimate of 12.77 million.

**Table 1.** Estimated population eligible for Bridge among US adults aged 65 years or older.

Measure	Estimate	95% CI	Notes
Base case: OSA 20%	10.93M	9.40M-12.47M	Conservative base case
Sensitivity: OSA 15%	11.39M	9.81M-12.98M	Lower OSA exclusion assumption
Sensitivity: no OSA adjustment	12.77M	11.00M-14.54M	Upper-bound estimate before OSA exclusion
Removed by diabetes exclusion	7.31M	5.65M-8.98M	Otherwise, eligible but excluded
Removed by modeled OSA exclusion	1.84M	—	20% of otherwise eligible adults with BMI ≥30
Base-case BMI 27 to <30	3.58M	2.45M-4.71M	BMI-band composition
Base-case BMI 30 to <35	4.02M	3.12M-4.91M	BMI-band composition
Base-case BMI ≥35	3.34M	2.62M-4.05M	BMI-band composition

OSA-adjusted confidence intervals reflect NHANES sampling uncertainty only and treat the modeled OSA exclusion rate as fixed.

Abbreviations: BMI, body mass index; OSA, obstructive sleep apnea.

In the base case, eligible beneficiaries were distributed across BMI bands: 3.58 million had BMI 27 to <30, 4.02 million had BMI 30 to <35, and 3.34 million had BMI ≥35.

These estimates exclude 7.31 million beneficiaries with diabetes, 0.57 million with MASH, and 1.84 million beneficiaries with OSA.

## Discussion

Nearly 11 million Americans 65 and older who indicated they have Medicare meet the eligibility criteria to receive GLP-1 medicines for obesity under the Bridge program. Many patients who are excluded from Bridge (eg, those with diabetes or OSA) may have access to GLP-1 medicines through Medicare Part D.

This estimate uses population-level health status and differs from an assessment of the size of the qualifying population under Bridge that used encounter data to estimate an eligible population of 3.8 million.^5^

This represents a sizable expansion of the potential market for obesity treatments and suggests that public outlays to fund the program, which is slated to run through December 31, 2027, could be considerable.

This study has limitations. The Medicare population may have changed since the 2017-2020 NHANES survey was fielded, and the dataset excludes individuals in long-term care. NHANES-derived estimates for heart failure and hypertension prevalence do not precisely match the Bridge inclusion criteria. PAD metrics are unavailable in NHANES, and OSA was modeled from published literature. In some cases, patients with CV risk factors may have certain GLP-1 medicines covered via the Medicare Part D program.

The impact of Bridge will depend on how broadly the medicines are used by the eligible population, making additional perspective on uptake and adherence critical to better understanding the potential public health and budgetary impacts of the program.

## Supplementary Material

qxag283_Supplementary_Data
