# Supplementary material for: Estimating the population eligible for Medicare's obesity-coverage demonstration
Source: Health Aff Sch. 2026 Oct 19;4(10):qxag283. doi: 10.1093/haschl/qxag283 (PMC13714906; doi:10.1093/haschl/qxag283)
Supplement: Supplementary file 1 [file qxag283_supplementary_data.zip › HA SCHOLAR-D-26-00568 Supplemental 9 21 26 .docx]

# Supplemental Tables

**Table S1. Weighted demographic characteristics**

| **Characteristic** | **Level** | **Overall estimate** | **Overall SE** | **Eligible estimate** | **Eligible SE** |
| --- | --- | --- | --- | --- | --- |
| Age, mean |  | 73.1 | 0.2 | 72.5 | 0.3 |
| BMI, mean |  | 29.4 | 0.2 | 33.3 | 0.2 |
| Sex, % | Female | 55.1 | 1.2 | 59.3 | 3.6 |
| Sex, % | Male | 44.9 | 1.2 | 40.7 | 3.6 |
| Race/ethnicity, % | Mexican American | 2.8 | 0.5 | 1.5 | 0.4 |
| Race/ethnicity, % | NH Asian | 4.0 | 0.8 | 1.3 | 0.4 |
| Race/ethnicity, % | NH Black | 8.6 | 1.5 | 9.2 | 1.6 |
| Race/ethnicity, % | NH White | 76.7 | 2.6 | 79.9 | 2.1 |
| Race/ethnicity, % | Other Hispanic | 4.7 | 0.7 | 5.3 | 0.9 |
| Race/ethnicity, % | Other/multiracial | 3.3 | 0.6 | 2.8 | 0.9 |

*Note. Overall refers to Medicare-covered adults aged ≥65 years. Eligible refers to adults eligible before modeled OSA exclusion, because OSA adjustment is modeled rather than observed at the person level. Values are mean or percent with standard errors.*

**Table S2. Unweighted analytic counts**

| **Measure** | **Unweighted n** |
| --- | --- |
| Analytic sample, Medicare age ≥65 with MEC weights | 2,056 |
| BMI ≥35 pathway | 333 |
| BMI ≥30 plus comorbidity pathway | 370 |
| BMI ≥27 plus comorbidity pathway | 661 |
| Gross eligible before exclusions | 883 |
| Excluded for diabetes | 354 |
| Excluded for MASH proxy | 27 |
| Eligible before modeled OSA exclusion | 502 |
| Eligible before OSA, BMI ≥30 | 347 |
| Primary OSA 20% modeled equivalent | 432.6 |

*Note. The primary OSA 20% row is a modeled unweighted equivalent, not an observed respondent count, because the OSA exclusion is applied fractionally.*

**Table S3. Missing data among Medicare-covered adults aged ≥65 years**

| **Variable** | **Missing, n** | **Total, n** | **Missing, %** |
| --- | --- | --- | --- |
| BMI | 292 | 2,270 | 12.9 |
| Hemoglobin A1c | 322 | 2,270 | 14.2 |
| Serum creatinine/eGFR | 386 | 2,270 | 17.0 |
| Measured blood pressure | 459 | 2,270 | 20.2 |
| Liver stiffness | 439 | 2,270 | 19.3 |
| CAP | 439 | 2,270 | 19.3 |
| AST | 392 | 2,270 | 17.3 |
| FAST score | 718 | 2,270 | 31.6 |

*Note. Missingness denominator includes all age ≥65 Medicare respondents before restriction to MEC-weighted analytic sample.*

**Table S4. Hypertension and antihypertensive medication diagnostics**

| **Diagnostic** | **Unweighted n** |
| --- | --- |
| Any antihypertensive agent | 1645 |
| ≥2 antihypertensive agents | 1087 |
| Uncontrolled BP | 711 |
| Old HTN proxy | 459 |
| Final Bridge HTN criterion | 365 |

*Note. Counts are among Medicare-covered adults aged ≥65 years.*

**Table S5. Comparison of drug-count vs ingredient-count hypertension definition**

| **Diagnostic** | **Value** |
| --- | --- |
| Old HTN proxy | 459 |
| ≥2 by drug count | 308 |
| ≥2 by ingredient count | 330 |
| Percent ≥2 by drug count | 67.1% |
| Percent ≥2 by ingredient count | 71.9% |

*Note. Counts are among respondents meeting the old hypertension proxy: uncontrolled BP plus self-reported BP medication use.*

**Table S6. MASH proxy diagnostics**

| **Diagnostic** | **Unweighted n** |
| --- | --- |
| Liver exam complete | 1687 |
| Valid liver elastography | 1552 |
| AST nonmissing | 1878 |
| FAST score nonmissing | 1552 |
| MASH proxy | 99 |

*Note. The MASH proxy uses FAST score ≥0.35 among respondents with valid liver elastography.*
